# A. V. M. A., 1900

**Published:** 1899-12

**Authors:** 


### A. V. M. A., 1900.

NO doubt before this issue of the JOURNAL reaches its readers the place of meeting for 1900 will have been decided by the votes of the Executive Committee. The Association can do much good at Minneapolis, Detroit, or Columbus, but as to which will bring the richest returns for our calling there may be the widest differences of opinion. To-day it looks as if our choice of Minneapolis would inure to the advantage of the largest number and be a fitting response to a large and faithful contingent of our membership.

Detroit is the storm-centre to-day of professional disturbances. The cause of higher veterinary education has received its death-knell for a time in that State. Her schools are closing their doors or demoralized in a supreme effort to gain personal advantages and supremacy in active competition for students by an attractive short term. Her State Association has not been powerful enough to sustain her higher grade schools or to mould their legislation in the interests of higher veterinary education now or prospectively. They have not been strong enough in State affairs to even obtain the appointment on this body of their deliberate selections. They are divided as to the character and kind of examination to be given to all applicants for registration under its provisions, and the meeting of our Association there might prove the panacea for all these professional ills.

Columbus stands out geographically as the place for this meeting to meet fully the views of those who feel that the belt of States between the East and the Middle West should be more strongly represented in our organization. As a college centre it has added its part to American Veterinary College history; in Association work it has had a varied history, and lags in the race to-day; in legislation it was one of the first to move; but its law has not been rigidly enough enforced to make it as yet the protection to her citizens that it should be. These are strong claims, and will no doubt be weighed well by the Executive Committee in arriving at a decision.

